# Evolution of Th2 responses: characterization of IL-4/13 in sea bass (*Dicentrarchus labrax* L.) and studies of expression and biological activity

**DOI:** 10.1038/s41598-017-02472-y

**Published:** 2017-05-22

**Authors:** Valentina Stocchi, Tiehui Wang, Elisa Randelli, Massimo Mazzini, Marco Gerdol, Alberto Pallavicini, Chris J. Secombes, Giuseppe Scapigliati, Francesco Buonocore

**Affiliations:** 1 Department for Innovation in Biological, Agro-food and Forest systems, University of Tuscia, Largo dell’Università snc, 05100 Viterbo (VT), Italy; 2 Scottish Fish Immunology Research Centre, School of Biological Sciences, University of Aberdeen, Aberdeen, AB24 2TZ UK; 3 Department of Life Sciences, University of Trieste, Via Giorgieri 5, 34127 Trieste (TS), Italy

## Abstract

Th2 immunity is a primary host defence against metazoan pathogens and two of the important cytokines involved in this immune response in mammals are IL-4 and IL-13. Recently the origin and evolution of Th2 immune responses have been investigated in fish where a molecule with relatedness to both IL-4 and IL-13 is present, termed IL-4/13. Different IL-4/13 paralogues (IL-4/13 A and IL-4/13B) exist in teleost fish. In this paper, we have focused on the IL-4/13 isoforms found in the European sea bass (*Dicentrarchus labrax* L.). Two tandem duplicated but divergent IL-4/13 A isoforms and one IL-4/13B are present, a unique situation compared to other teleosts. These genes were studied in terms of their *in vitro* and *in vivo* transcript levels after different treatments and their biological activities after production of the recombinant isoforms. The results show that the presence of these three paralogues is associated with different activities, both in terms of their expression profiles and the ability of the proteins to modulate the expression of immune genes in head kidney leukocytes. It is clear that the initiation and control of type-2 responses in seabass is complex due to the presence of multiple IL-4/13 isoforms with overlapping but distinct activities.

## Introduction

Helper T cells, T lymphocytes expressing the cell surface molecule CD4, can be subdivided into Th1 and Th2 cells and the cytokines they produce are therefore known as Th1-type and Th2-type cytokines. Th2-type cytokines include interleukin-4 (IL-4) and interleukin-13 (IL-13), which were first identified in mammals about 30 years ago^1^. IL-4 and IL-13 have similar activity in relation to the immune system, but they are also involved in other physiological processes, like pregnancy, foetal development, some brain functions and in the pathogenesis of atopy and asthma^1–5^. IL-4 is well known as a T-cell derived growth factor and an immunoglobulin switch factor^6, 7^ and it acts on a wide range of both haematopoietic and non-haematopoietic cells^8^. IL-13 can elicit most of the known IL-4 activities, but it also shows unique effector functions that distinguish this cytokine from IL-4^9^. For example, in a recent paper on allergic responses mediated by IL-4 and IL-13, it was evidenced that IL-4 mediates many specific functions, including fine-tuning of the Th2 response through its ability to initiate, perpetuate or shut off the allergic response through the activation of multiple signalling pathways (STAT6 and IRS-2), whilst IL-13 preferentially drives the development of the disease pathological features manifested by non-bone marrow–derived cells^10^. Moreover, other researchers demonstrated that in asthma pathogenesis IL-4 can induce similar lung pathology to IL-13, but independent from IL-13 and that IL-13Ralpha1 regulates IL-4-induced responses^11^. IL-4 can induce similar lung pathology to IL-13, but independent from IL-13, and that IL-13Rα1 regulates the differential responses of IL-4 and IL-13^11^. IL-4 and IL-13 share about 25% amino acid sequence identity in mammals, and are short four α-helix glycoproteins^12^ whose genes are tandemly organized and located on human chromosome 5^13^. Type-2 inflammatory processes initiated by IL-4 and IL-13^1^ are fundamental for immune defence against helminth parasites^14, 15^. IL-4 and IL-13 are recognized on the target cell surface by receptor heterodimers composed of three possible subunits (IL-4Rα, IL-13Rα1 and the common γ-chain, γC). IL-4 interacts with both the type I receptor composed of IL-4Rα and γC and the type II receptor of IL-4Rα and IL-13Rα1, whereas IL-13 binds only to the type II receptor. IL-13 can also interact with the IL-13Rα2 subunit that seems to act as a “decoy” receptor for IL-13^16^. Signal transducer and activator of transcription 6 (STAT-6) and insulin receptor substrate 2 (IRS-2) are considered the primary molecules involved in IL-4 and IL-13 signalling after binding to a specific cell surface receptor^1^.

The origin and evolution of Th2 immune responses have been studied during the last years^17^, with the aim to understand when this essential component of the adaptive immune system first emerged in vertebrates. The receptor subunits found in mammals for IL-4 and IL-13 have been identified throughout the jawed vertebrates^17^ and two copies of each have been cloned recently in salmonids^18^. An interesting feature is that although IL-4 and IL-13 are present in birds, clear orthologues are missing in other vertebrates^17^. However, in fish two IL-4/IL-13 related genes were identified in pufferfish (*Tetraodon nigroviridis*)^19^ and in zebrafish (*Danio rerio*)^20, 21^ in two different loci, one close to the RAD50 gene and the other to the KIF3A gene, respectively. It has been proposed that the duplication of these IL-4/13 genes arose after the third round (3R) of whole genome duplication (WGD) that occurred in the teleost fish ancestor^21^. A new nomenclature was proposed, where the gene adjacent to RAD50 was termed IL-4/13A and the other adjacent to KIF3A was termed IL-4/13B^21^. In the spotted gar (*Lepisosteus oculatus*), a 2R bony fish, a single IL-4/13 gene is present between KIF3A and RAD50^17^ and at least two IL-4/13 related genes have been identified in the elephant shark (*Callorhinchus milii*), a 2R cartilaginous fish^22^.

IL-4/13 isoforms have been identified in salmonids and their expression studied in depth^23, 24^. A cell line expressing IL-4/13B has been obtained from carp (*Cyprinus carpio*) to investigate *in vitro* Th2 fish immune responses^25^. Finally, a possible Th2 inflammatory process has been discovered in coho salmon (*Oncorhynchus kisutch*) after a disease outbreak due to the salmon louse (*Lepeophtheirus salmonis*)^26^ and recombinant IL-4/13 molecules have been produced in zebrafish^27^, carp^28^, rainbow trout^24^, Japanese pufferfish^29^ and goldfish^30^ to investigate the fish Th2-like regulatory mechanisms.

In this paper we present the identification and molecular characterization of three IL-4/13 isoforms in European sea bass (*Dicentrarchus labrax* L.). Uniquely in sea bass two IL-4/13A isoforms are present, adjacent to each other in the genome, and have relatively low homology (37.9% identity/58.6% similarity) giving the potential for further functional divergence of IL-4/13 genes in this species. Transcriptional analysis of the three isoforms was undertaken in healthy fish, and after *in vitro* and *in vivo* stimulation. The production of the three molecules as recombinant proteins allowed investigation of the biological activity of these important type-2 cytokines in sea bass. It is apparent that the three sea bass IL-4/13 isoforms have different functions in terms of their expression patterns and the ability of the proteins to modulate immune-related genes in target cells. Thus, the control of type-2 responses in fish species such as sea bass is complex and demonstrates that some fish species have expanded further their Th2 cytokine repertoire.

## Results

### The nucleotide sequence analysis of sea bass IL-4/13 isoforms

Three IL-4/13 sequences (accession numbers KJ818332 for IL-4/13A1; KJ818333 for IL-4/13A2 and KJ818331 for IL-4/13B) have been identified in a sea bass gill transcriptome^31^ and successively confirmed by cloning from gill cDNA (data not shown). Each cDNA sequence had an in frame stop codon before the main open reading frame (ORF) and a 3′-untranslated region (UTR). They encoded three putative proteins of 144, 142 and 148 amino acids, with predicted signal peptides of 23, 23 and 20 amino acids, and 3, 1 and 4 potential N-glycosylation sites, respectively (see Figures S1–S3). Two genes show potential polyadenylation signals and all have multiple ATTTA motifs in the 3′-UTR (see Figures S1–S3).

### The structure and synteny analysis of sea bass IL4/13 genes

**Figure 1:**
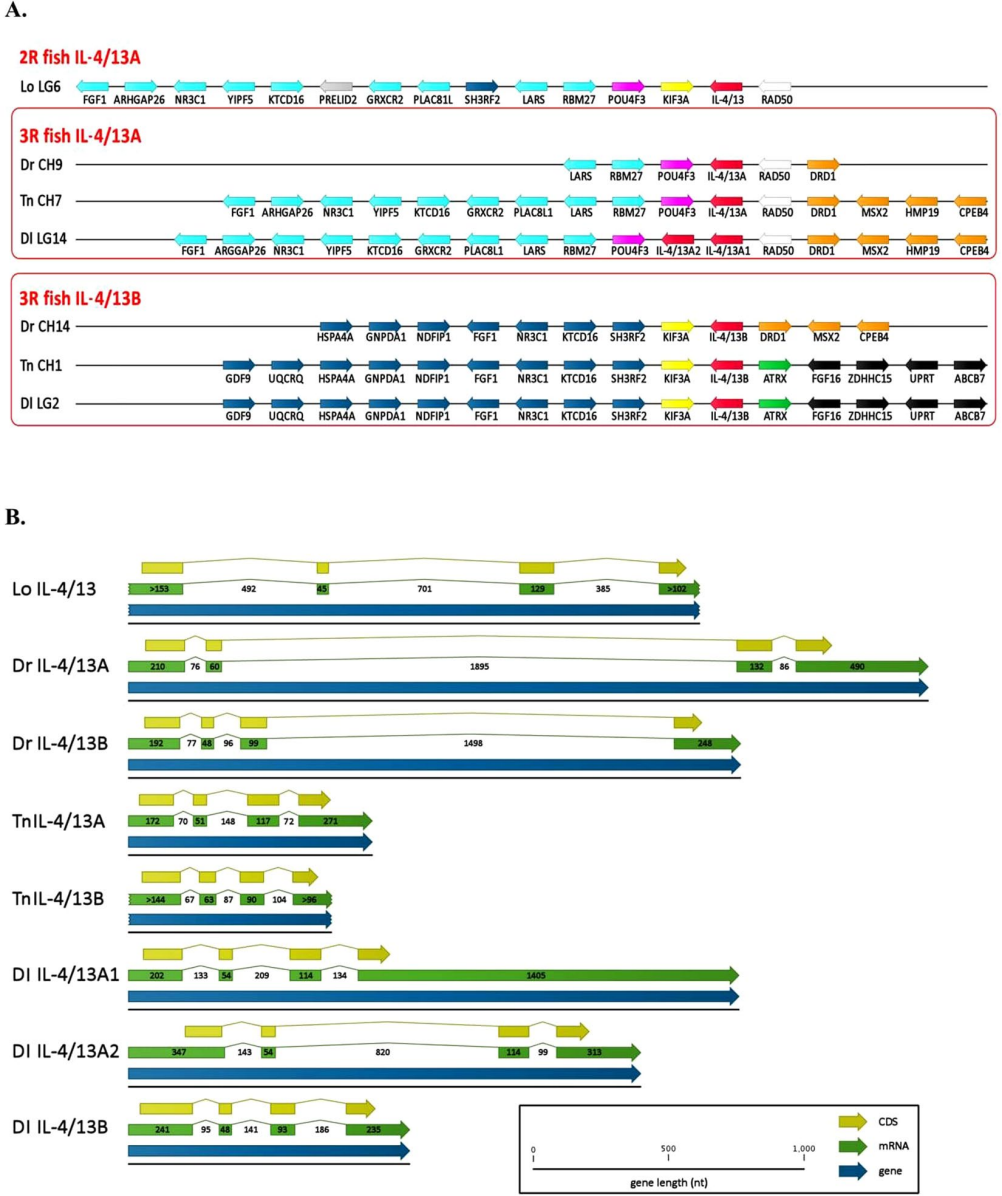
Panel A. *Gene synteny of IL-4/13 loci across bony fishes*. The unique locus of the 2R spotted gar *Lepisosteus oculatus* and the two duplicated loci (**A** and **B**) of the 3 R zebrafish *Danio rerio*, green spotted pufferfish *Tetraodon nigroviridis* and European sea bass *Dicentrarchus labrax* are shown. Evolutionarily relevant genes located at the 5′ and at the 3′ of the IL-4/13 loci are evidenced and their strand orientations are indicated with arrows. The immediately flanking (POU4F3/RAD50 and KIF3A/ATRX) and neighbouring genes at each side of the 2R/3R fish IL-4/13 loci are differently coloured using the European sea bass genomic organization as a reference. Panel B. *Exon/intron organization of IL-4/13 genes in bony fishes*. Gene, mRNA and CDS annotations are shown. Uncertain exon boundaries are indicated by broken arrows. Numbers present on the sequences indicate exon and intron length (bp) Lo: *Lepisosteus oculatus*; Dr: *Danio rerio*; Tn: *Tetraodon nigroviridis*; Dl: *Dicentrarchus labrax*”.

The structure of the European sea bass IL-4/13 genes corresponding to the three isoforms identified from the sea bass gill transcriptome is fully consistent with those previously reported for other teleost Th2 interleukins^17, 21, 24^. Considering their position on the identified sea bass genomic loci, two IL-4/13 isoforms have been identified as A1 and A2, that are adjacent and linked to RAD50, and the other as B, that is linked to KIF3A (Fig. 1, Panel A). The three genes consist of four exons and three introns of highly variable length (Fig. 1, Panel B, and accession numbers KP096353-5). The coding sequence spans all four exons, with the initial ATG codon located on exon 1 and the STOP codon located on exon 4. Like all the other teleost IL-4/13 genes, all introns are in phase 0. Noteworthy is the 3′-UTR of the IL-4/13A1 mRNA which, at over 1 Kb, is by far the longest 3′-UTR reported so far in a Th2 cytokine in fish (Fig. 1, Panel B).

The synteny analysis identified a highly conserved block of orthologous genes between the 3R teleost sea bass (*D. labrax*) and green spotted pufferfish (*T. nigroviridis*) in both the A and B loci, with local gene duplication of the IL-4/13A in the sea bass as the only remarkable difference. The two loci of the zebrafish (*D. rerio*) display a peculiar gene organization: while locus A maintains a block of 6 conserved genes (including IL-4/13A), locus B only presents one of the two genes which flank IL-4/13B in other Euteleostei, KIF3A, which appears to keep its association with seven other genes. ATRX on the other hand is not associated with any IL-4/13 locus and the other flanking gene is curiously DRD1, which is duplicated in zebrafish and is found associated to RAD50 in locus A in sea bass and pufferfish. Besides DRD1, two other genes usually associated with locus A in Euteleostei (MSX2 and CPEB4) are located in locus B in zebrafish.

A single IL-4/13 locus is present in the 2R teleost spotted gar (*L. oculatus*). Although KIF3A and RAD50 are the two genes which flank IL-4/13 (an hybrid situation compared to Euteleostei), POU4F3 is located next to KIF3A, and it is associated with all the genes present in the locus A of Euteleostei.

### The amino acid sequence analysis of sea bass IL-4/13 isoforms

**Figure 2:**
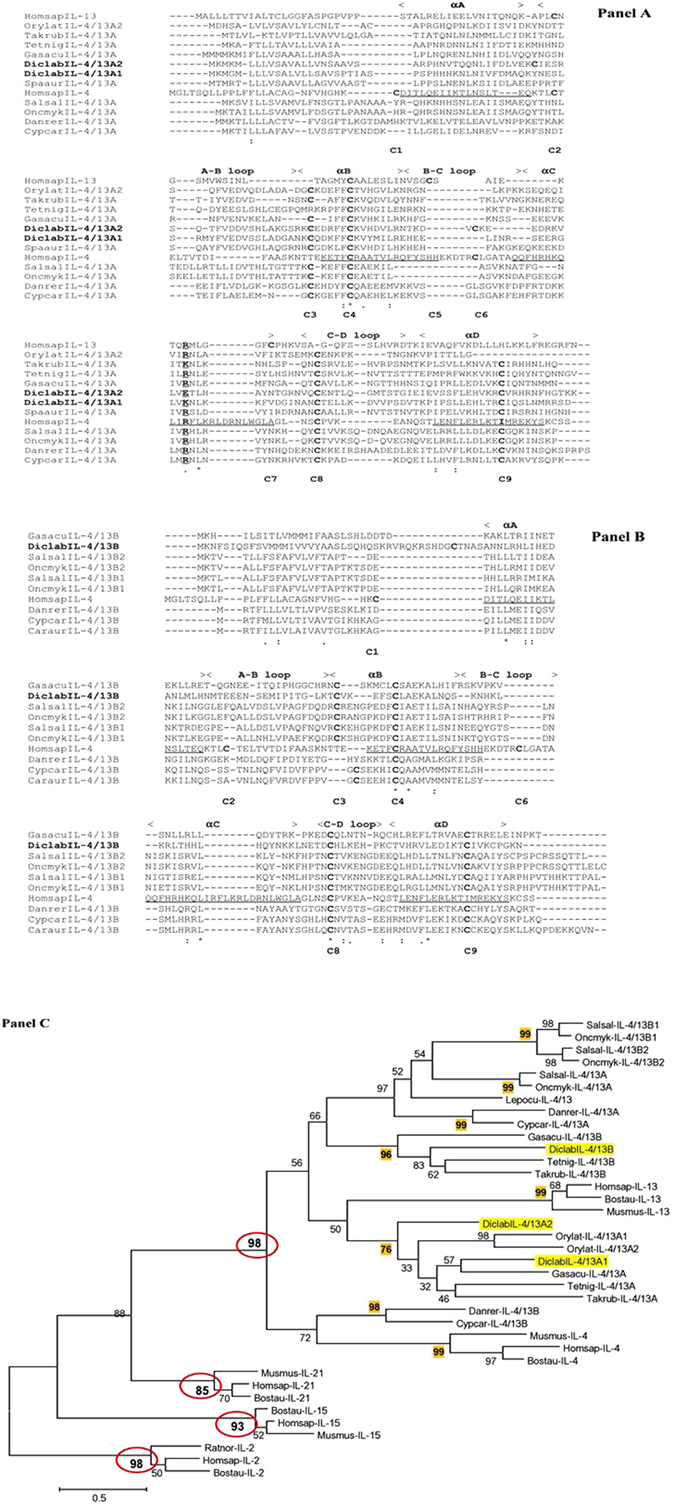
Panel A and Panel B. *Amino acid sequence alignment of the predicted sea bass IL-4/13 isoforms with selected IL-4/13 molecules*. The conserved amino acids are indicated with an “_*_” below the sequences, while “.” and “:” show amino acids with conserved physical and/or chemical properties. The position of the nine cysteine residues found in the different sequences is highlighted below the alignment and shown in bold along the sequences. The four α-helices and loop regions known for human IL-4 are shown above the alignment and the amino acids involved in the α-helices underlined in the human IL-4 sequence. Panel C. *Phylogenetic tree analysis of fish IL-4/13 molecules with mammalian IL-4/IL-13 molecules and other closely related γ-chain cytokines IL-2, IL-15 and IL-21*. The phylogenetic tree was constructed using amino acid multiple alignments and the neighbour-joining method within the MEGA7 program. The percentage of replicate trees in which the associated taxa clustered together in the bootstrap test (10,000 replicates) was shown next to the branches. *Lepisosteus oculatus* (spotted gar) IL-4/13 was predicted from chromosome LG6. The bootstrapping values that support lineage-specific groupings are highlighted with a yellow background.

The amino acid sequence identity and similarity of sea bass IL-4/13 isoforms with other known teleost IL-4/13 molecules is shown in Table 1. With regard to sea bass IL-4/13A1, the highest amino acid identity was found with IL-4/13A from three-spined stickleback (39.9%) followed by medaka (34.9%), whereas sea bass IL-4/13A2 showed the highest identity with green spotted pufferfish IL-4/13A. Sea bass IL-4/13B has the highest identity with the pufferfish IL-4/13B (31.6%), followed by IL-4/13B from three-spined stickleback (26.8%). Overall the identity values are quite low, even between the sea bass IL-4/13A1 and IL-4/13A2 (only 37.9%), and this aspect is an indication of the very fast evolutionary diversification within these molecules belonging to the class-I helical cytokine family^32^. A multiple alignment with different IL-4/13 cytokines from fish was assembled together with IL-4 and IL-13 from human (see Fig. 2, Panel A and Panel B). In most teleost IL-4/13A molecules four cysteine residues were found, as seen in human IL-4 and IL-13, but the residues are in different positions compared to the human sequences^17^. The only cysteine residue that is conserved in all sequences is the one identified as C4 (see Fig. 2, Panel A and Panel B). Sea bass IL-4/13A1 contains four cysteine residues and, as predicted for zebrafish IL-4/13A, they should form two disulphide bridges^21^. In sea bass IL-4/13A2 six cysteine residues are present, whereas in the sea bass IL-4/13B sequence seven cysteine residues have been found. Another conserved residue in most sequences is the arginine in the αC helix that is an important residue for the binding of human IL-4 with its receptor (IL-4Rα)^33^. Sea bass IL-4/13 isoforms do not show the same amino acid in that position but IL-4/13A1 and IL-4/13A2 have a polar and charged residue (lysine and glutamic acid, respectively) and IL-4/13B a polar residue (histidine). Despite these differences between teleost IL-4/13 sequences, in phylogenetic tree analysis the sea bass IL-4/13 molecules cluster with all the known fish IL-4/13 sequences, together with the mammalian IL-4 and IL-13 sequences, with high bootstrap support (98%). Within this cluster the sea bass IL-4/13A1 and IL-4/13A2 sequences form a clade with other neoteleostean IL-4/13A sequences (see Fig. 2, Panel C) and sea bass IL-4/13B with the neoteleostean IL-4/13B sequences, with high bootstrap support. Similarly, known protacanthopterygian IL-4/13A and IL-4/13B sequences (i.e. salmon/trout) form separate clades, as do the otocephalan sequences (i.e. carp/zebrafish), hinting at lineage specific evolution of the IL-4/13 isoforms in teleost fish.

**Table 1:** Percentage of amino acid identity and similarity of sea bass IL-4/13 cytokines with selected IL-4/13 sequences from teleosts.

	Amino Acid Identity			Amino Acid Similarity		
	A1	A2	B	A1	A2	B
Dicentrarchus labrax IL-4/13A1		**37.9**	17.5		**58.6**	31.6
Dicentrarchus labrax IL-4/13A2	**37.9**		16.4	**58.6**		29.8
Dicentrarchus labrax IL-4/13B	17.5	16.4		31.6	29.8	
Salmo salar IL-4/13 A	24.7	24.5	24.7	38.3	41.5	38.0
Oncorhynchus mykiss IL-4/13 A	26.3	25.8	**27.6**	40.7	42.3	37.6
Danio rerio IL-4/13 A	19.0	22.8	17.1	38.1	42.0	34.7
Danio rerio IL-4/13B	18.4	21.5	18.6	31.3	38.0	32.7
Cyprinus carpio IL-4/13 A	21.2	23.0	17.8	41.7	43.4	33.1
Cyprinus carpio IL-4/13B	19.2	26.2	17.2	29.7	39.6	34.4
Oryzias latipes IL-4/13A1	**34.9**	28.9	19.9	50.0	**51.3**	34.5
Oryzias latipes IL-4/13A2	29.8	29.5	16.0	46.4	47.3	29.6
Takifugu rubripes IL-4/13 A	31.1	27.0	18.3	52.3	45.4	28.8
Takifugu rubripes IL-4/13B	22.4	21.9	**31.6**	40.8	39.0	**49.3**
Tetraodon nigrodiviris IL-4/13 A	29.5	**35.4**	20.6	57.5	**53.7**	36.5
Tetraodon nigrodiviris IL-4/13B	20.9	22.5	25.7	36.1	41.1	**47.4**
Gasterosteus aculeatus IL-4/13 A	**39.9**	**34.2**	21.7	52.0	47.9	33.1
Gasterosteus aculeatus IL-4/13B	11.5	19.9	**26.8**	23.0	37.1	**43.3**
Carassius auratus IL-4/13B	18.2	24.5	17.4	30.7	37.4	34.3

The highest values are highlighted in bold.

### Basal expression of sea bass IL-4/13 isoforms

**Figure 3:**
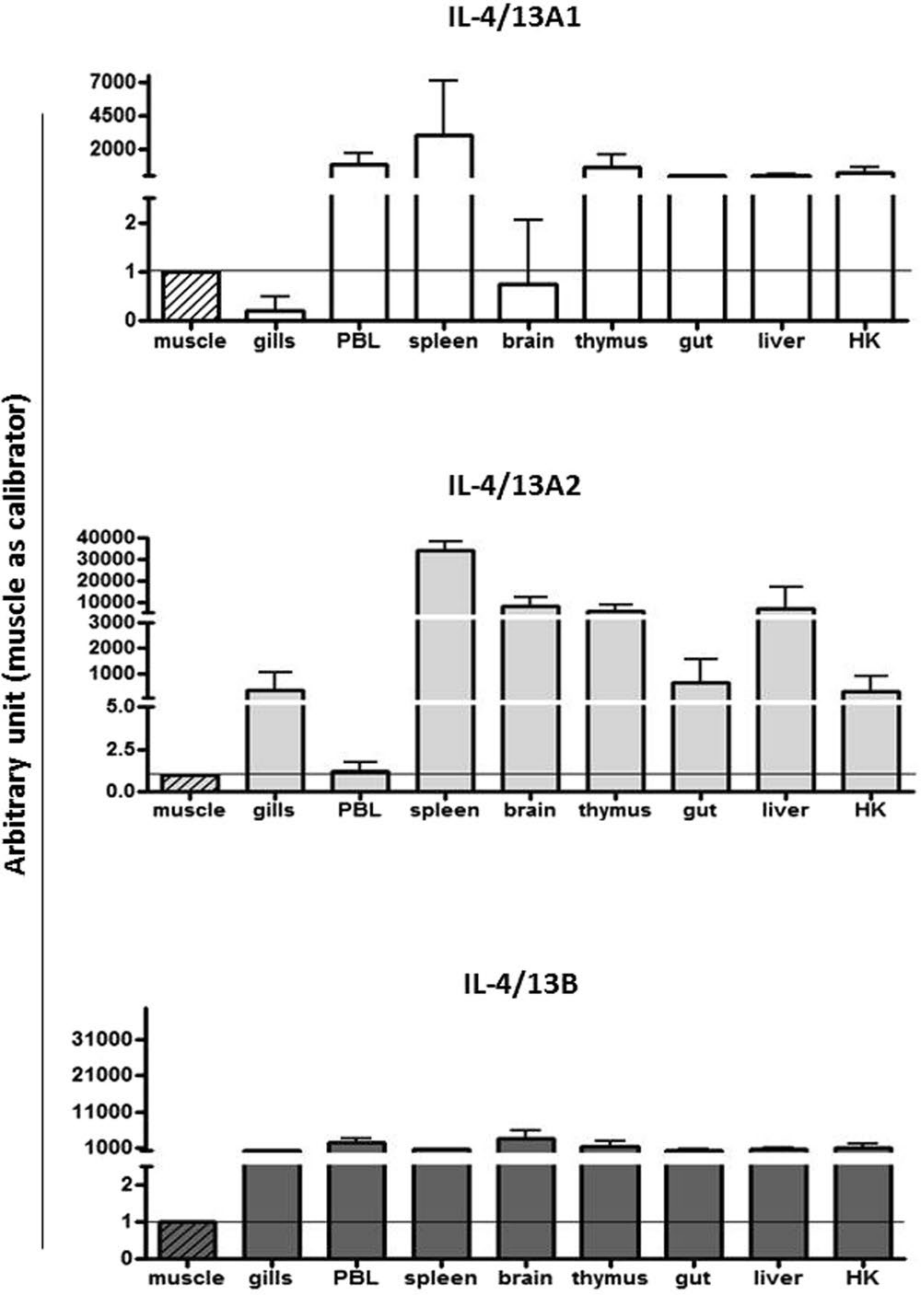
The basal expression of sea bass IL-4/13 isoforms in different tissues. Sea bass IL-4/13 mRNA levels were first normalised to that of 18S rRNA in the same tissue after real-time PCR analysis, and expressed as arbitrary unit using the expression level in the muscle as a calibrator (1 unit). Data were expressed as the mean + SD of four healthy sea bass juveniles.

The constitutive expression of the three sea bass IL-4/13 isoforms has been analysed in 9 different tissues of healthy fish (see Fig. 3). Sea bass IL-14/13A1 is highly expressed in spleen, followed by PBL, thymus, HK, gut and liver, with very low transcript levels found in gills. IL-4/13A2 is also highly expressed in spleen, followed by thymus, liver and brain, with very low transcript levels detectable in PBL. In contrast, the sea bass IL-4/13B isoform was expressed at almost the same level in all examined tissues.

### Expression analyses of sea bass IL-4/13 isoforms *in vitro* and *in vivo*

**Figure 4:**
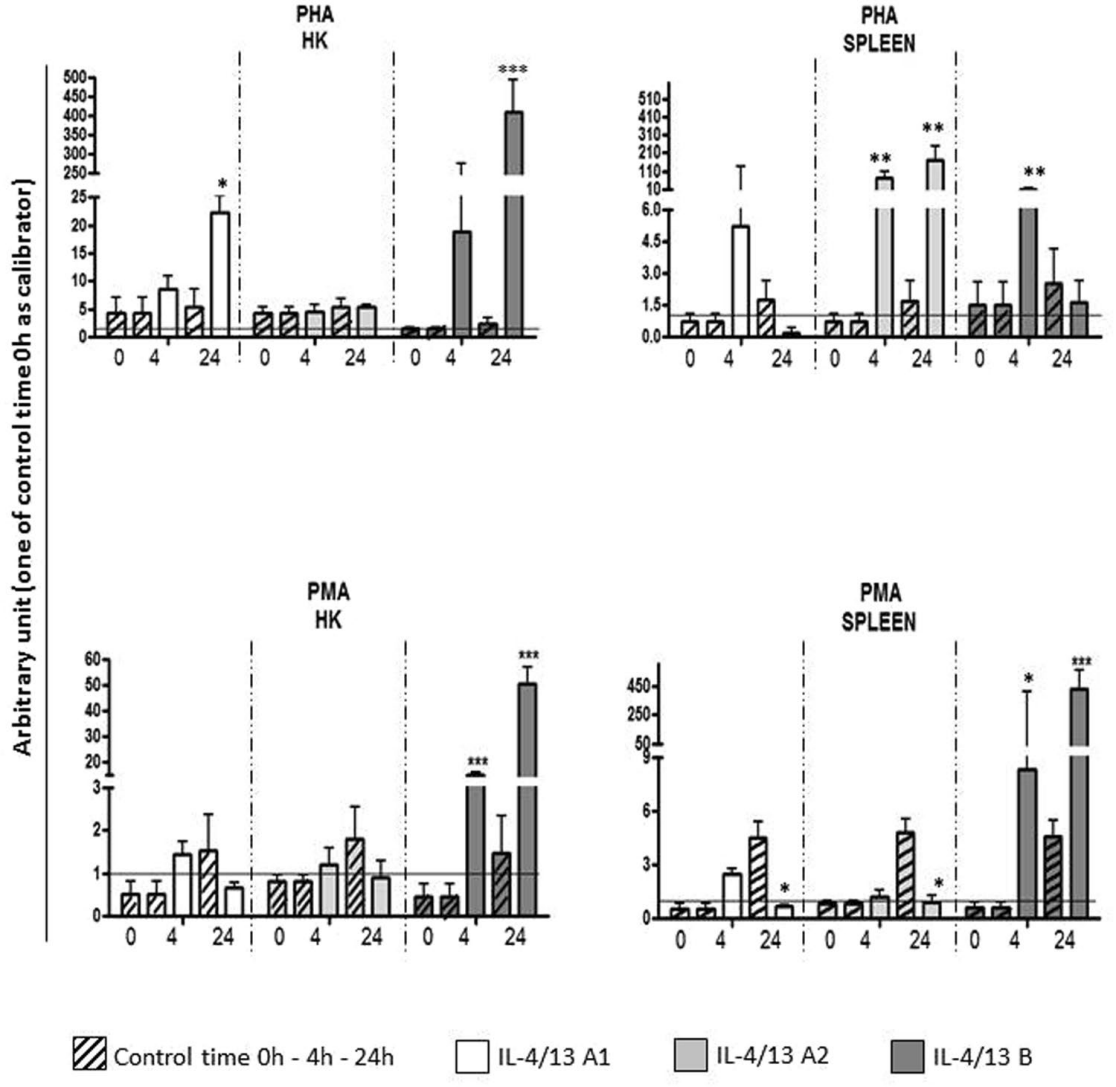
The expression of sea bass IL-4/13 isoforms after in vitro stimulation with PHA and PMA. The mRNA levels of sea bass IL-4/13 isoforms were normalised to that of 18S rRNA in the same samples after real-time PCR analysis of HK and spleen leukocytes stimulated with L-15 medium (control) or with 10 µg/ml of PHA or with 1 µg/ml PMA for 4 h and 24 h, and expressed as arbitrary unit against the non-stimulated 0 h control. Data were expressed as the mean + SD. *p < 0.05 with respect to the time 0 control; ^**^p < 0.01 with respect to the time 0 control; ***p < 0.001 with respect to the time 0 control; N = 4.

To investigate the modulation of expression of sea bass IL-4/13 isoforms we performed *in vitro* stimulation of leukocytes from HK and spleen with PHA, a T cell mitogen, and PMA, separately (Fig. 4). PHA stimulated the expression of IL-4/13A1 and IL-4/13B in HK at 24 h. In contrast, in spleen IL-4/13A2 was up-regulated both at 4 and 24 h and IL-4/13B at 4 h. PMA stimulation also produced an increase of IL-4/13B expression in HK, which was significant at both 4 h and 24 h. In spleen a more highly significant increase of IL-4/13B expression was seen at 24 h.

**Figure 5:**
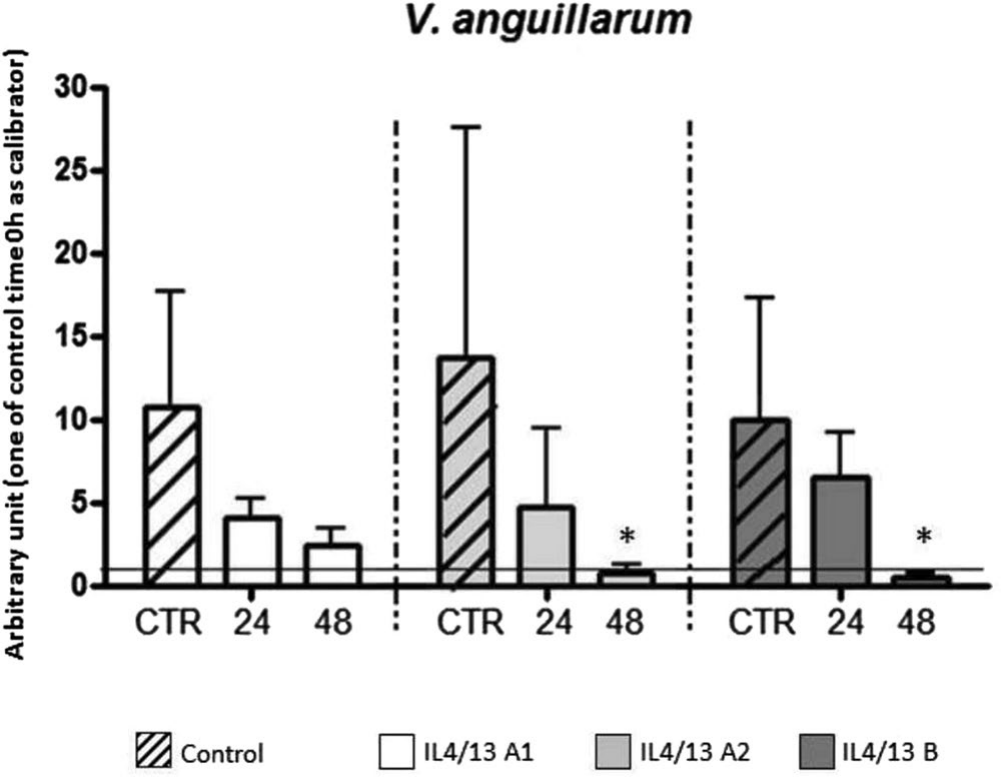
Sea bass isoforms IL-4/13 expression analysis after vaccination against *Vibrio anguillarum*. Sea bass IL-4/13 isoforms mRNA levels were expressed as a ratio relative to rRNA 18S in the same samples after real-time PCR analysis of HK leukocytes of four fish vaccinated against *Vibrio anguillarum*. Data were expressed as the mean + SD. *p < 0.05 with respect to the time 0 control.

To gain some information on the role of the IL-4/13 isoforms in the sea bass immune response following vaccination, we detected their gene expression after administration of a commercial vaccine against *Vibrio anguillarum*, one of the most common bacterial vaccines used in aquaculture. After vaccination down-regulation of all three IL-14/13 isoforms was detected in HK after 24 h and 48 h (Fig. 5), although only the 48 h effects were statistically significant for IL-4/13A2 and IL-4/13B.

### Production of the recombinant sea bass IL-4/13 isoforms

Proteins of the expected size of 15.4 kDa (recombinant (r) IL-4/13A1), 15.3 kDa (rIL-4/13A2) and 16.3 kDa (rIL-4/13B) were induced by IPTG stimulation of transformed BL21 cells, and purified under denaturing conditions with extensive washing in 1% Triton X-100 buffer to remove LPS (Figure S4). The purified proteins were refolded *in vitro* and re-purified under native conditions; denaturants and other contaminants were removed by extensive washing of the purification column.

### *In vitro* biological activity of recombinant sea bass IL-4/13 isoforms

**Figure 6:**
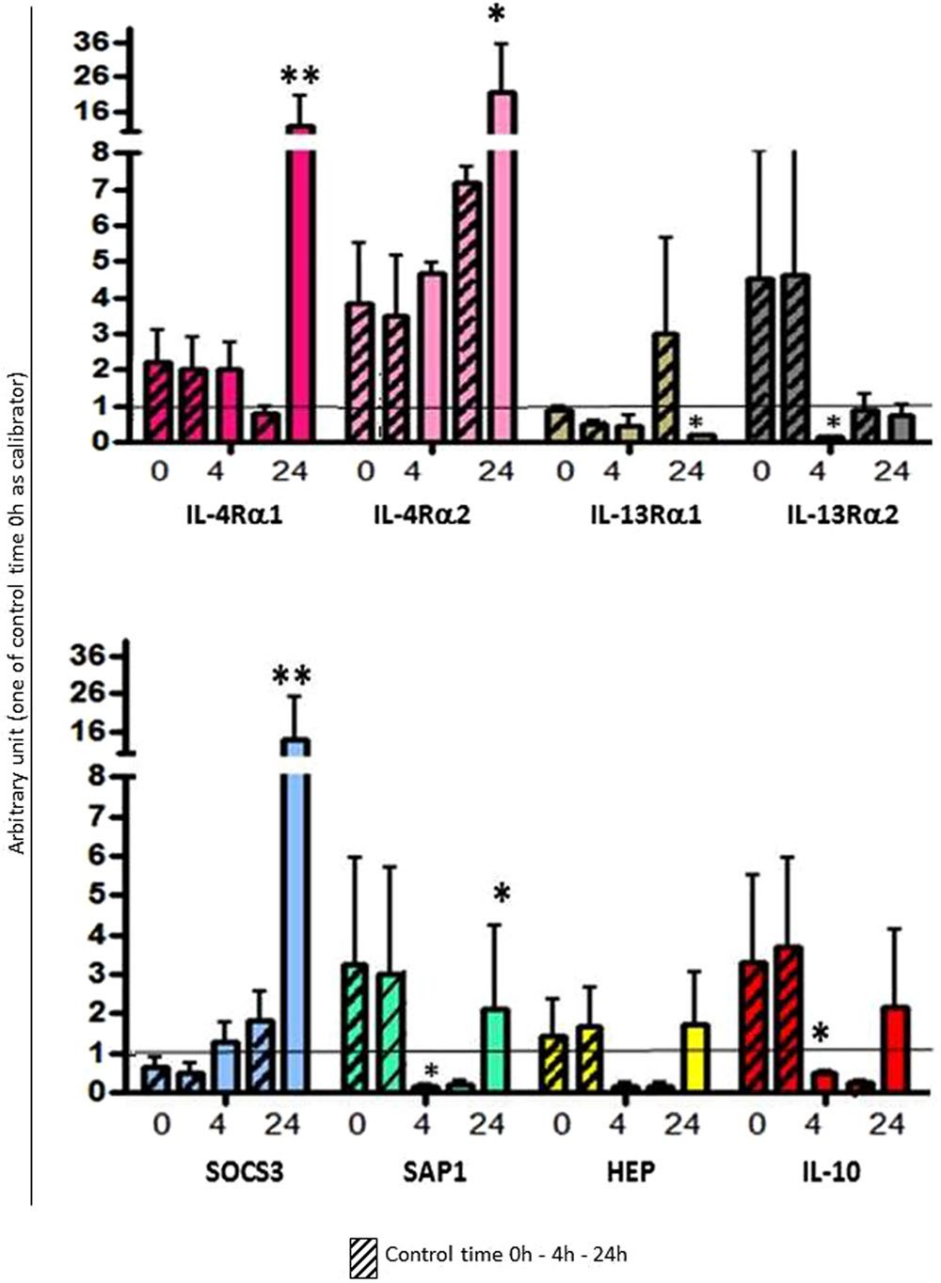
Biological activity of sea bass recombinant IL-4/13A1. The expression level of genes coding for IL-4Rα1, IL-4Rα2, IL-13Rα1, IL-13Rα2, SOCS3, SAP1, HEP and IL-10 was determined in HK leukocytes after stimulation with the sea bass recombinant IL-4/13A1. Transcription values were expressed as a ratio relative to 18S rRNA in the same samples. The quantitative PCR amplification was performed in PCR arrays and each point represents the mean + SD of cells from 4 individual fish. *p < 0.05 with respect to the time 0 control; **p < 0.01 with respect to the time 0 control.

**Figure 7:**
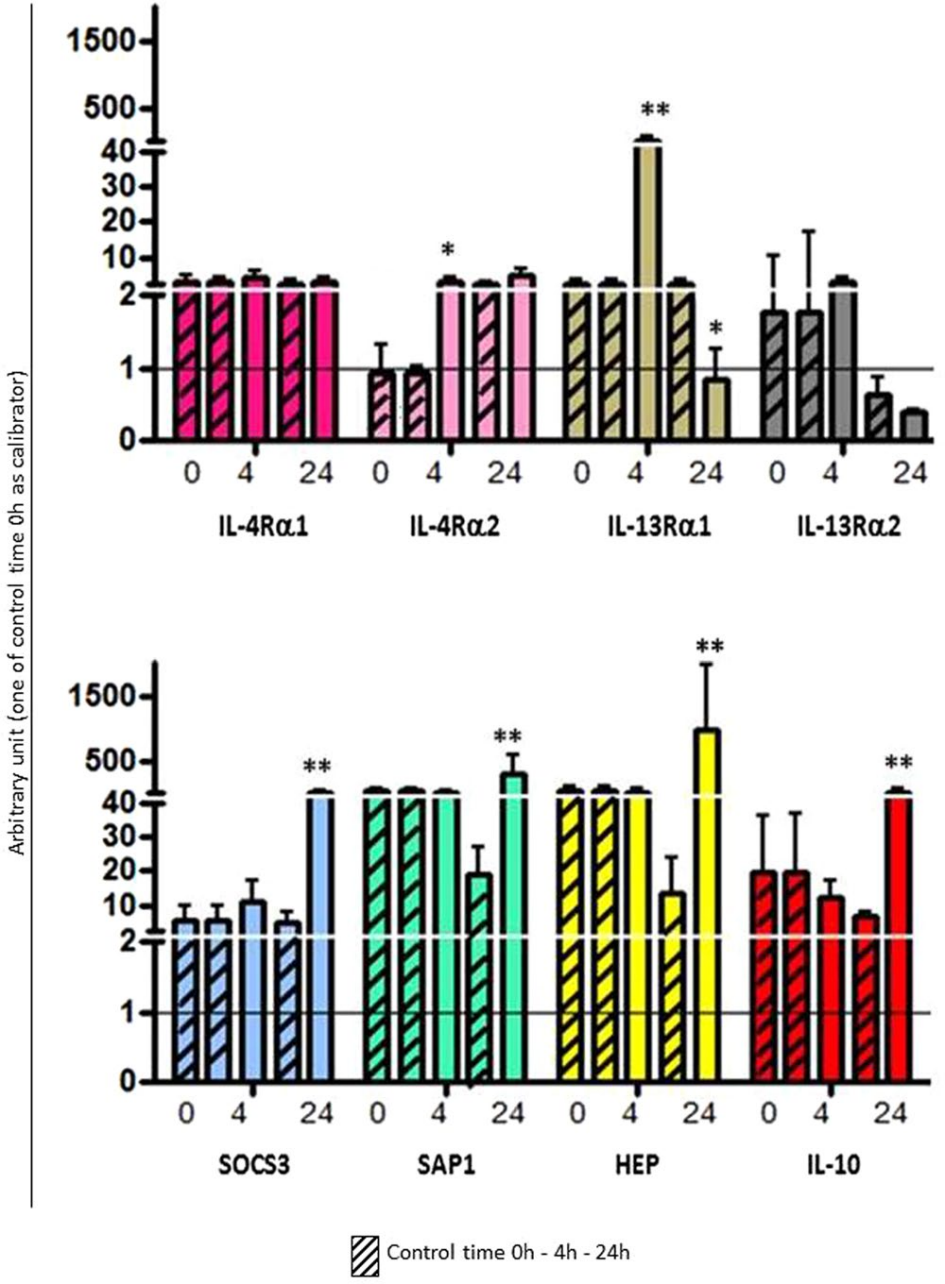
Biological activity of sea bass recombinant IL-4/13A2. The expression level of genes coding for IL-4Rα1, IL-4Rα2, IL-13Rα1, IL-13Rα2, SOCS3, SAP1, HEP and IL-10 was determined in HK leukocytes after stimulation with the sea bass recombinant IL-4/13A2. Transcription values were expressed as a ratio relative to 18S rRNA in the same samples. The quantitative PCR amplification was performed in PCR arrays and each point represents the mean + SD of cells from 4 individual fish. *p < 0.05 with respect to the time 0 control; **p < 0.01 with respect to the time 0 control.

**Figure 8:**
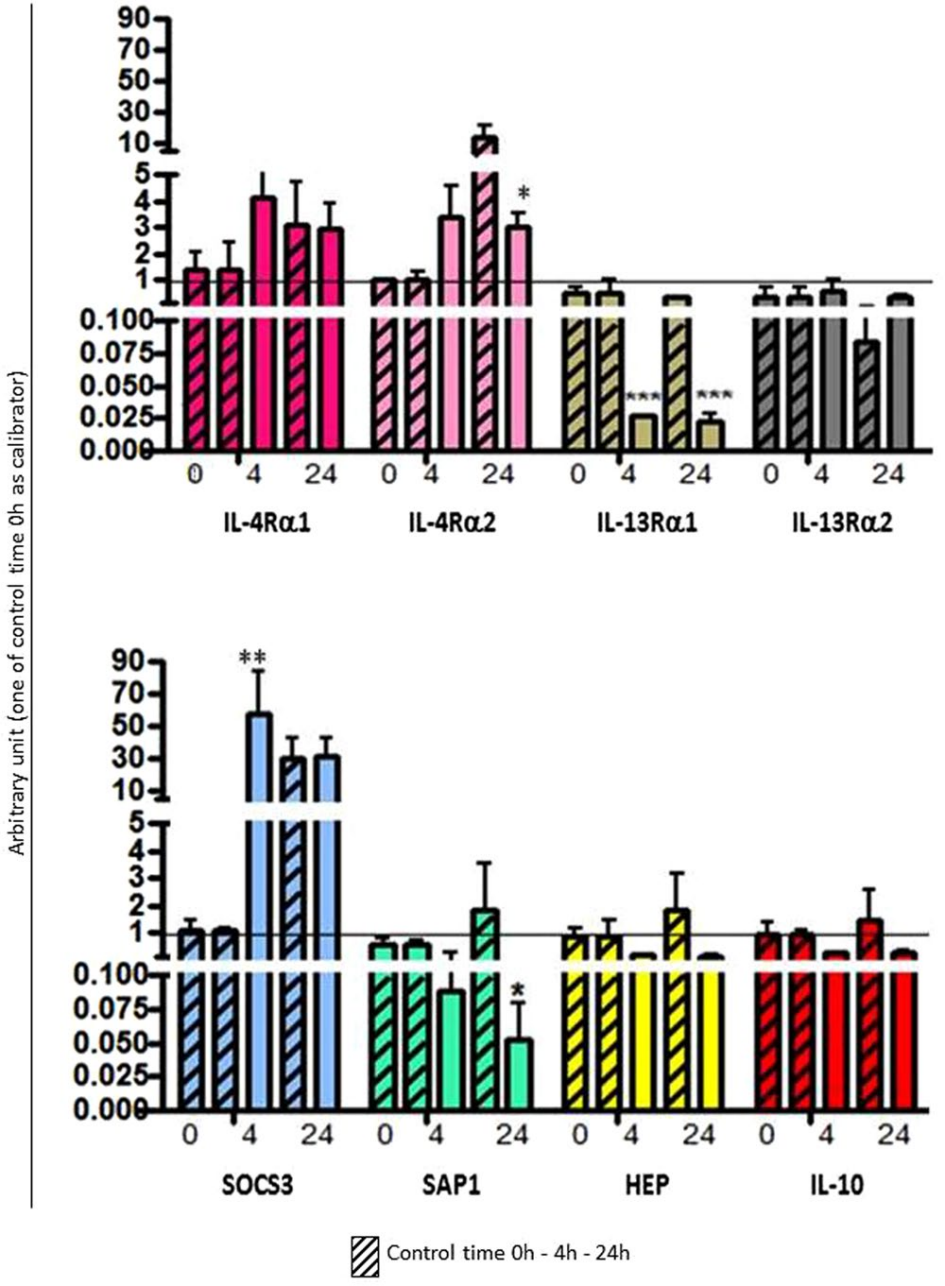
Biological activity of sea bass recombinant IL-4/13B. The expression level of genes coding for IL-4Rα1, IL-4Rα2, IL-13Rα1, IL-13Rα2, SOCS3, SAP1, HEP and IL-10 was determined in HK leukocytes after stimulation with the sea bass recombinant IL-4/13B. Transcription values were expressed as a ratio relative to 18S rRNA in the same samples. The quantitative PCR amplification was performed in PCR arrays and each point represents the mean + SD of cells from 4 individual fish. *p < 0.05 with respect to the time 0 control; **p < 0.01 with respect to the time 0 control; ***p < 0.001 with respect to the time 0 control.

The biological activity of the recombinant sea bass IL-4/13 isoforms on HK leukocytes has been investigated (Figs 6, 7 and 8) after 4 h and 24 h stimulation, using the same concentration previously selected for trout recombinant IL-4/13 isoforms^24^. To be sure that there was no or very little LPS contamination in the purified recombinant proteins, a *Limulus* amoebocyte lysate assay was performed (Bio Whittaker) and the bacterial endotoxin content at the used concentration of IL-4/13 was calculated to be 1.5 ng/ml. This quantity is considerably less than the minimum amount needed to induce inflammation in fish HK leukocytes^34^. The effects of the recombinant IL-4/13 isoforms on sea bass leukocytes were studied in relation to the expression of several important immune-related genes, including: IL-4/13 receptors (IL-4Rα1, IL-4Rα2, IL-13Rα1, IL-13Rα2); acute phase protein and antimicrobial peptide genes (SAP1 and hepcidin, HEP); a cytokine (IL-10) and a SOCS gene (SOCS3) involved in down-regulating inflammatory effects. The recombinant IL-4/13A1 (Fig. 6) up-regulated the expression of both IL-4Rα isoforms, SOCS3 and SAP1 after 24 h. It also down-regulated the expression of IL-13Rα2, SAP1 and IL-10 at 4 h and IL-13Rα1 at 24 h. The recombinant IL-4/13A2 (Fig. 7) also up-regulated SOCS3 and SAP1 at 24 h and down-regulated IL-13Rα1 expression at 4 h post-stimulation, but in addition up-regulated IL-4Rα2 and IL-13Rα1 at 4 h and hepcidin and IL-10 at 24 h. Lastly, the recombinant IL-4/13B (Fig. 8) also up-regulated SOCS3 but at 4 h rather than 24 h, although no other positive effects were seen. In addition it down-regulated IL-13Rα1 at 4 h and 24 h, but in contrast to the IL-4/13A isoforms it also down-regulated IL-4Rα2 and SAP1 at 24 h.

## Discussion

Type-2 immune responses are involved in fundamental aspects of the adaptive immune system, like anti-helminth immunity, modulation of type-1 driven autoimmune diseases and neutralization of toxins. In mammals, two important cytokines that are activated and define these responses are interleukin-4 (IL-4) and IL-13^12^. Over the last decade the origin and evolution of these immune responses has started to be investigated due to the identification of ancestral molecules related to IL-4 and IL-13 in other vertebrates. In teleost fish, two types of genes related to IL-4 and IL-13 exist, named IL-4/13A and IL-4/13B. They have been identified in several species and it is evident that they arose by the duplication of an ancestral IL-4/13 gene as a consequence of a whole genome duplication event that took place in this lineage^17, 21^.

In this paper we have characterised the IL-4/13 isoforms in European sea bass (*Dicentrarchus labrax* L.) and show that uniquely there are two IL-4/13A isoforms present in addition to IL-4/13B. The two IL-4/13 isoforms are clearly the result of a tandem gene duplication event, and are next to each other in the genome and associated with RAD50 as seen with other teleost IL-4/13A genes. These three isoforms share low homology, i.e. IL-4/13A1 and IL-4/13A2 share 37.9% amino acid identity with each other (Table 1), and 17.5% and 16.4% identity with IL-4/13B, respectively. Even lower homology is seen to the IL-4 and IL-13 sequences, such that the sea bass IL-4/13A1 shares only 18% amino acid identity with human IL-4. It is well known that type-2 cytokines are fast evolving genes, as seen in mammals where, for example, human and cow IL-4 have only 40% of amino acid identity. Despite this low amino acid identity there is a clear association of all the IL-4, IL-13, IL-4/13A and IL-4/13B isoforms in the phylogenetic tree, which form a separate clade to over gamma-chain cytokines. Interestingly there are clear signs of lineage specific evolution of the IL-4/13 isoforms in teleost fish, with the IL-4/13A and IL-4/13B genes clustering separately for the neoteleosts, salmonids and cyprinids respectively. The gene organization is also considered lineage specific in teleost fish, in terms of the size of exon 3^24^. The sea bass isoforms conform to this hypothesis in having exon 3 larger in IL-4/13As vs IL-4/13B, and are identical to the stickleback homologues, in contrast to salmonids where the opposite is true. Comparing the two IL-4/13A genes in more detail, the main differences between IL-4/13A1 and IL-4/13A2 are the presence of an unusually large 3′-UTR in the IL-4/13A1 isoform, and a bigger (~x4) intron 2 in IL-4/13A2 (Fig. 1B).

The constitutive expression of the IL-4/13A and IL-4/13B isoforms in teleost fish is often species-specific and in sea bass it was evidenced that IL-4/13B is expressed at a similar level in all tissues, whereas IL-4/13A1 and IL-4/13A2 are differentially expressed in the examined organs, with both IL-4/13A isoforms highly expressed in the spleen (Fig. 3). Curiously the relative expression in gills and PBL was the opposite for IL-4/13A1 and IL-4/13A2, hinting at a functional divergence between these two isoforms in sea bass. One possible reason for the differential basal expression of the sea bass IL-4/13 isoforms is that they are expressed in different cell types that differ in abundance in each of the examined tissues. In rainbow trout and Atlantic salmon the highest IL-4/13A expression was found in gills, thymus, blood and skin^23, 24^, whereas the highest IL-4/13A expression was in gills in pufferfish^19^ and in gut, kidney, spleen, skin, gills and PBL in zebrafish^27^. However, it should be noted that the expression was determined by semi-quantitative PCR in pufferfish and zebrafish. In goldfish the highest expression of both IL-4/13A and IL-4/13B was found in heart, spleen, brain and kidney^30^. In salmonids two isoforms of IL-4/13B are present due to the salmonid WGD event^24^. The expression of trout IL-4/13B1 is highest in thymus and gills, whereas IL-4/13B2 transcript levels are highest in gills and muscle^24^, and again hints at sub-functionalisation when multiple isoforms are present within a species, as seen with other immune genes in fish^35^.

The potential also exists for the three sea bass IL-4/13 isoforms to be differentially modulated after stimulation, and this was tested using cultured leukocytes *in vitro* and by vaccination *in vivo*. Following *in vitro* stimulation with PHA and PMA of HK and spleen leukocytes (Fig. 4), the IL-4/13B isoform was highly induced. This has also been seen in rainbow trout, where PHA highly up-regulates the expression of the IL-4/13B isoforms and, to a lesser extent, IL-4/13A in HK cells^24^. Curiously PHA stimulation had differential effects on IL-4/13A isoforms expression in sea bass, up-regulating IL-4/13A1 in HK but IL-4/13A2 in spleen. An up-regulation of IL-4/13A expression in spleen and HK was also seen in pufferfish after *in vivo* injection of a mixture containing ConA/PMA/PHA, although it should be noted that the analysis was made by semi-quantitative RT-PCR^19^. That known T cell mitogens can up-regulate these IL-4/13 genes suggests that some of the isoforms are expressed in fish T cells, in addition to a potential innate lymphoid cell contribution. After sea bass vaccination with a *Vibrio anguillarum* oral vaccine (Fig. 5), both IL-4/13A2 and IL-4/13B were significantly down-regulated and this could indicate that the HK response at this time is switched to other defensive pathways, such as a Th17 response to combat extracellular bacteria or that vaccination induced an emigration of leukocytes expressing these cytokines from this tissue. In trout the expression of IL-4/13 isoforms was investigated in HK after infection with *Yersinia ruckeri*, another Gram-negative bacterial pathogen of fish that causes enteric redmouth disease (ERM)^24^, and there was also a significant down-regulation of IL-4/13B1 at 48 h post-challenge. However, in this model, prior (injection) vaccination prevented this decrease and was considered a potential marker of an effective vaccination.

The biological activity of the three IL-4/13 isoforms has been investigated using the produced recombinant IL-4/13 proteins after *in vitro* stimulation of HK leukocytes (Figs 6, 7 and 8). Two possible receptor genes for IL-4Rα and IL-13Rα were identified in sea bass in a gill transcriptome^31^ and their expression was found to be modulated in different ways by the recombinant IL-4/13 isoforms. IL-4/13A1 and IL-4/13A2 up-regulated the expression of both IL-4Rα receptors, although only at 24 h post-stimulation for IL-4/13A1 and only at 4 h for IL-4/13Rα2 in the case of IL-4/13A2. In contrast IL-4/13B down regulated IL-4Rα2 expression at 24 h. The IL-4/13 isoforms generally inhibited the expression of the IL-13Rα genes, with the exception of IL-13Rα1 at 4 h. In rainbow trout^24^ rIL-4/13A and rIL-4/13B2 have been shown to modulate the expression of these receptors, where they increase expression of IL-4Rα1/2 and IL-13Rα2, especially with rIL-4/13A in the latter case, but decrease the expression of IL-13Rα1. Without knowing the potential ligand-receptor interactions that may occur with three IL-4/13 isoforms in sea bass it is difficult to interpret the likely impact on responsiveness and signalling in target cells of these effects in receptor expression. Nevertheless it is clear that there is a complex regulation of IL-4/13 signalling and that the different isoforms have different roles and different kinetics. IL-4/13A1 and IL-4/13A2 also up-regulated SAP1 at 24 h, and hepcidin in the case of IL-4/13A2. In contrast IL-4/13B had the opposite effect on SAP1 at this time. This could evidence that the IL-4/3 A isoforms have a role in the activation of the innate immune system. Both of these genes were up-regulated in trout by both isoforms^24^, showing that differences are apparent in isoform function between fish species. Lastly, all three isoforms up-regulated the anti-inflammatory gene SOCS3, although with different kinetics between the IL-4/13A isoforms (24 h) vs IL-4/13B (4 h), but had variable effects on IL-10 expression ranging from early down-regulation (IL-4/13A1), late up-regulation (IL-4/13A2), or no effect (IL-4/13B). Such data could suggest a general anti-inflammatory role of these type-2 cytokines but again with subtle differences apparent between the isoforms. This is in agreement with mammals, where IL-4 and IL-13 are known to down-regulate pro-inflammatory genes^36^, and also agrees with the trout data where both genes are up-regulated by rIL-4/13A and rIL-4/13B, although primarily by the former for IL-10 as seen in sea bass with IL-4/13A2 (but not for IL-4/13A1).

In conclusion, the type-2 cytokines identified in sea bass show a pattern of biological activities that resemble their counterparts in mammals and reinforce the view that Th2-immunity is active in teleost fish. However, the presence of three paralogues gives the opportunity to modulate this immune response in more subtle ways in terms of the kinetics of the induced effects and possibly in terms of the receptor usage/target cells. That IL-4/13B is highly induced after stimulation with T cell mitogens suggests that at least this cytokine is related to Th2 responses, and relevant for analysis of adaptive immunity in sea bass. In addition, the expanded IL-4/13A system in sea bass gives a unique opportunity to gain a deeper understanding of the evolution of immune regulation, in this case in the context of type-2 responses in fish. Clearly we still have a lot to learn about the variability of cytokine function in different species.

## Materials and Methods

### Cloning and sequence analysis of sea bass IL-4/13 isoforms

Three nucleotide sequences related to different IL-4/13 isoforms (accession numbers KJ818331-3) were identified after the analysis of a sea bass gill transcriptome^31^. The sequences have been confirmed by PCR cloning of the entire coding region (data not shown) from sea bass gill cDNA obtained as described previously^37^. The sea bass IL-4/13 amino acid sequences were compared with counterparts in other species with the EMBOSS Pairwise Alignment tool. The IL-4/13 sequences were analysed for the presence of a signal peptide using SignalP software^38^, and of N-linked glycosylation sites, with the NetNGlyc 1.0 Server. Alignment of the sea bass IL-4/13 amino acid sequences to selected IL-4/13 molecules from other species was carried out using MEGA 4.1 Software^39^. A phylogenetic tree was constructed using the alignment and the neighbour joining method within the MEGA7 program^40^ and the evolutionary distances were computed using the JTT matrix-based method, with all ambiguous positions removed for each sequence pair.

### Synteny and gene organization analysis of sea bass IL-4/13 isoforms

The annotated genome data from the spotted gar *Lepisosteus oculatus* (v. LepOcu1), the zebrafish *Danio reri*o (v. GRCz10) and the pufferfish *Tetraodon nigroviridis* (v. TETRAODON 8.0) were retrieved from Ensembl (http://www.ensembl.org/index.html). The genome sequence of *Dicentrarchus labrax* was downloaded from NCBI Genomes (GCA_000689215.1 seabass_V1.0)^41^. Ensembl gene annotations were manually checked to assess synteny in the genomic region neighbouring the European sea bass IL-4/13A1, IL-4/13A2 and IL-4/13B loci. The relative position and orientation of different genes for locus A and for locus B was taken into account.

Exon/intron organizations were retrieved from Ensembl annotations, whenever available (*D. rerio*), inferred from the alignment of available ESTs with the genome (*T. nigroviridis*, locus A) or manually annotated by the combination of significant BLAST similarity with other type 2 cytokines of bony fish (the e-value threshold was set at 1 × 10^−3^), the presence of uninterrupted ORFs and compatible donor and acceptor splicing sites, as predicted by NNSPLICE v. 0.9^42^.

The annotation of the European sea bass loci was performed by aligning the *de novo* assembled mRNAs to the reference genome with MUSCLE^43^. Sea bass IL-4/13 mRNA sequences were obtained, as described before, from the gill transcriptome and *de novo* assembled as described by Nunez-Ortiz *et al*.^31^. The exon/intron junctions and the gene boundaries were refined by NNSPLICE v. 0.9^42^ and by the alignment of raw sequence data to the reference genome by the large gap read mapping tool included in the Genomics Workbench v.8 (Qiagen, Hilden, Germany), with mismatch/insertion/deletion costs set to 3, and length and similarity fraction parameters set to 0.98 and 0.75, respectively.

### The analyses of basal expression of sea bass IL-4/13 isoforms

To investigate the basal expression levels of sea bass IL-4/13 isoforms, four sea bass juveniles were sampled and different tissues (muscle, gills, peripheral blood leukocytes (PBL), spleen, brain, thymus, gut, liver, head kidney (HK)) obtained as described before^37^. Total RNA was isolated from each tissue separately with TRIsure (Bioline), resuspended in DEPC treated water and used for reverse-transcription real-time quantitative PCR without pooling the tissue samples coming from the different fish. Controls for the presence of DNA contamination were performed using β-actin primers that bracket an intron (see Table 2). For reverse transcription, the BioScript RNase H minus (Bioline) enzyme was used with the protocol described previously^44^. The expression level of IL-4/13 transcripts was determined with a Mx3000P real-time PCR system (Stratagene) as described before^44^. Specific PCR primers were designed for the amplification of about 200 bp products from the three IL-4/13 isoforms (see Table 2). A relative quantitation was performed, comparing the levels of the target transcript (the IL-4/13 isoforms) to a reference transcript (calibrator, the tissue with the lowest IL-4/13 expression, in this case the muscle). A normalizer target (18S ribosomal RNA) was included to correct for differences in total cDNA input between samples. The results are expressed as the mean ± SD of the results obtained from the four fish, with duplicate samples performed for each fish.

**Table 2:** Primers used for expression analysis and production of recombinant proteins.

Gene	Primers Sequence 5′-3′(forward, FW, and reverse, RV)	Accession number
β-actin	ATGTACGTTGCCATCC (FW) GAGATGCCACGCTCTC (RV)	AJ493428
18S ribosomal RNA	CCAACGAGCTGCTGACC (FW, real-time PCR) CCGTTACCCGTGGTCC (RV, real-time PCR)	AY831388
IL-4/13A1	ATGGTGCAAACAAATGTCAGGATAA (FW, real-time PCR) TGACGTCTGAAGGGACCACAT (RV, real-time PCR)	KJ818332
IL-4/13A2	GCAGCAGAAAATGTGAGGATCG (FW, real-time PCR) GATCTCTATGCCTGTACTTGTGTCATTC (RV, real-time PCR)	KJ818333
IL-4/13B	TCATGAAGACGCAAATCTGATGT (FW, real-time PCR) CGAGACAGGAGAACTCTTTCACACA (RV, real-time PCR)	KJ818331
IL-4/13A1	AGTCCTTCTCCTCATCATCACAAGAAC (FW, recombinant) ATCGCTTCGTCTCATGTTCAAGC (RV, recombinant)	KJ818332
IL-4/13A2	CGTCCTCATAACGTTACTCAGCAAAAC (FW, recombinant) TTTTTTGGTCCCATGAAAGTTTCTGTGC (RV, recombinant)	KJ818333
IL-4/13B	GCATCACTCTCACAACATCAAAGCA (FW, recombinant) GTTTTTCCCAGGACACTTGACAATACATG (RV, recombinant)	KJ818331
IL-4Rα1	GGTTTGGGCTTTATGCCGTCC (FW, real-time PCR) CACTGTACCAACCACTTCGAG (RV, real-time PCR)	KT852977
IL-4Rα2	GACCAGGTGAGATCTTGGC (FW, real-time PCR) GCACAGGAGGTTCCAGTGTA (RV, real-time PCR)	KT809424
IL-13Rα1	GAGAATATGATGAAAGTCCCCTACG (FW, real-time PCR) CTTGAATATTGCTGAAGGATCTGG (RV, real-time PCR)	KT809426
IL-13Rα2	CTACATGATGTGCAATTGGGAAAG (FW, real-time PCR) TGTTGGTATCAGGGGCCC (RV, real-time PCR)	KT809425
SOCS3	GAGAGTGGCTTCTACTGGGG (FW, real-time PCR) GATCAGCTTGAGGACGCAGTC (RV, real-time PCR)	KP642762
SAP1	TCTGGCCACACCATCCGC (FW, real-time PCR) GGGTGTAGTAATGTTTGATCCTCC (RV, real-time PCR)	KP642763
HEP	TGCAGTGGCCGTCGTGC (FW, real-time PCR) CAATTGCAGCAAAAGCGACAGC (RV, real-time PCR)	DQ131605
IL-10	ACCCCGTTCGCTTGCCA (FW, real-time PCR) CATCTGGTGACATCACTC (RV, real-time PCR)	AM268529

### *In vitro* and *in vivo* sea bass IL-4/13 isoforms expression analyses

The *in vitro* IL-4/13 isoform expression was studied after stimulation of HK and spleen leukocytes isolated from four sea bass juveniles (100 g of weight). Cells were cultured in L-15 medium (Gibco) containing 10% FCS, adjusted to a concentration of 1 × 10^5^ cells/ml and incubated at 22 °C with 10 µg/ml of leucoagglutinin from *Phaseolus vulgaris* (PHA, Sigma) or with 1 µg/ml of phorbol 12-myristate 13-acetate (PMA, Sigma) for 4 h and 24 h. The samples used as control were incubated with L-15 medium alone.

Total RNA was isolated with TRIsure (Bioline) as described above and used for real-time quantitative PCR without pooling the samples coming from the different fish. The primers and the real-time PCR conditions were the same as described above, except that the calibrator for this experiment was the time 0 control. The results were expressed as the mean ± SD of the results obtained from four fish and the differences from the control were considered significant if p < 0.05 using two-way ANOVA analysis followed by the Bonferroni’s post-hoc test.

The *in vivo* IL-4/13 isoforms expression analysis was performed from fish vaccinated with a commercial vaccine designed against the sea bass bacterial pathogen *Vibrio anguillarum* (Aquavac Vibrio Oral, Merck). The vaccination was performed on 50 sea bass individuals (30–40 g). The fish were fed at a feeding rate of 1.5% of fish biomass with a commercial pellet diet (Skretting) supplemented over 10 days with Aquavac Vibrio Oral (the delivery was performed over 15 days with 5 days off in the middle, to achieve a 0.2 ml final concentration of antigen as suggested by the manufacturers). The control group of 50 sea bass individuals (30–40 g) was fed with the commercial diet only. For gene expression analyses, 7 fish/group/time point (0, 24 h and 48 h after the end of the vaccination procedure) were sampled and from each individual fish the HK was removed. RNA extraction, cDNA preparation and real time PCR analysis were performed as described above using as calibrator the time 0 control.

### Production of the recombinant sea bass IL-4/13 isoforms

The coding regions of the predicted mature peptides of the three sea bass IL-4/13 isoforms were amplified from mixed cDNA samples (from gills, gut and HK) using primers detailed in Table 2 with the Q5 high fidelity enzyme (New England Biolabs, UK). The amplified products were cloned in a pET vector (Invitrogen) and confirmed by sequencing. The constructs encode identical mature peptides with a His-tag (MGSHHHHHHHHS) added at the N-terminus for easy purification. Thus, the recombinant seabass IL-4/13 isoforms are 133 aa, 131 aa and 140 aa, with a calculated molecular weight of 15.4 kDa, 15.3 kDa and 16.3 kDa, and a theoretical pI of 7.23, 8.58 and 9.14, for IL-4/13-A1, -A2 and -B, respectively. Following transformation of the plasmid into BL21 Star (DE3) competent cells (Invitrogen) and the induction of recombinant protein production, a first purification was performed under denaturing conditions. Successively we undertook a refolding process, a re-purification under native conditions, a SDS-PAGE analysis of the purified proteins and a quantification of the protein concentration as described in detail previously^45–47^. The refolding buffer contained 50 mM Tris-HCl (pH 7.5), 10% glycerol, 0.5 M arginine monohydrochloride, and 5 mM 2-mercaptoethanol. The purified proteins were desalted in desalting buffer (DSB) (PBS containing 50% glycerol) using PD-10 Desalting Columns (GE Healthcare). After sterilization with a 0.2 µm filter, the recombinant proteins were aliquoted and stored at -80 °C, ready for the *in vitro* stimulation of sea bass cells.

### *In vitro* biological activity of recombinant sea bass IL-4/13 isoforms

The *in vitro* biological activity of recombinant IL-4/13 isoforms was studied using leukocytes isolated from HK of four sea bass juveniles (100 g of weight). Cells were cultured in L-15 medium (Gibco) containing 10% FCS, adjusted to 1 × 10^5^ cells/ml and incubated at 22 °C for 4 h and 24 h with 200 ng/ml of each recombinant isoform separately. The cell control samples were stimulated with DSB buffer alone.

Total RNA was isolated with TRIsure (Bioline) as described above and used for real-time quantitative PCR without pooling the samples coming from the different fish.

The biological activity of the recombinant isoforms was monitored by studying the regulation of the transcription level of different target genes shown to be modulated in trout by IL-4/13 and identified from the sea bass gill transcriptome^31^. These included: IL-4 receptor α1 (IL-4Rα1), IL-4 receptor α2 (IL-4Rα2), IL-13 receptor α1 (IL-13Rα1), IL-13 receptor α2 (IL-13Rα2), suppressor of cytokine signalling 3 (SOCS3), serum amyloid P1 (SAP1), hepcidin (HEP), and interleukin-10 (IL-10). The sea bass IL-4Rα1 amino acid sequence showed ~51% identity with IL-4Rα1 identified in rainbow trout^18^, whereas sea bass IL-4Rα2 had ~52% identity with the rainbow trout homologue^18^; the two sea bass IL-4Rα shared ~65% amino acid identity. The sea bass IL-13Rα1 amino acid sequence had ~50% identity with IL-13Rα1beta identified in rainbow trout^13^, whereas sea bass IL-13Rα2 had ~50% identity with the rainbow trout homologue^48^; the two sea bass IL-13Rα shared ~40% amino acid identity. Specific PCR primers (Table 2) were designed for the amplification of products (~200 bp) from the conserved region of all selected genes. Real-time PCR conditions were the same as described above with the only exception that the annealing temperature was 52 °C; the calibrator for this experiment was the time 0 control. The results were expressed as the mean ± SD of the results obtained from four fish and the differences from the control were considered significant if p < 0.05 using the two-way ANOVA analysis followed by the Bonferroni’s post-hoc test.

### Statistical analysis

The data of all real-time PCR experiments were expressed as the mean ± SD of the results obtained from four fish. The statistical analysis was performed using the software GraphPad Prism 4 (two-way ANOVA) and Sigma Plot (Bonferroni test). Data were considered significant if p < 0.05.

### Use of experimental animals

All fishes were handled complying with the Guidelines of the European Union Council and of the Ethical Committee of the Tuscia University (Prof. Giuseppe Scapigliati, Prof. Nicola Lacetera, Prof. Nicla Romano) for the use of live animals. All experimental animal protocols were approved by the Ethical Committee of the Tuscia University (Prof. Giuseppe Scapigliati, Prof. Nicola Lacetera, Prof. Nicla Romano).

## Electronic supplementary material

Supplementary information
