# Supplementary material for: Evolution of Th2 responses: characterization of IL-4/13 in sea bass (Dicentrarchus labrax L.) and studies of expression and biological activity
Source: Sci Rep. 2017 May 22;7:2240. doi: 10.1038/s41598-017-02472-y (PMC5440397; doi:10.1038/s41598-017-02472-y)
Supplement: Supplementary file 1 — Supplementary information [file 41598_2017_2472_MOESM1_ESM.pdf]

Evolution of Th2 responses: characterization of IL-4/13 in sea bass (*Dicentrarchus labrax* L.) and studies of expression and biological activity

Valentina Stocchi[a1], Tiehui Wang[a2], Elisa Randelli[1], Massimo Mazzini[1], Marco Gerdol[3], Alberto Pallavicini[3], Chris J. Secombes[2], Giuseppe Scapigliati[1], Francesco Buonocore[1*]

[1]Department for Innovation in Biological, Agro-food and Forest systems, University of Tuscia, Largo dell'Università snc, 05100 Viterbo (VT), Italy

[2]Scottish Fish Immunology Research Centre, School of Biological Sciences, University of Aberdeen, Aberdeen AB24 2TZ, UK

[3]Department of Life Sciences, University of Trieste, Via Giorgieri 5, 34127 Trieste (TS), Italy

[a]These authors contributed equally to the paper

*Corresponding author at: Department for Innovation in Biological, Agro-food and Forest systems, University of Tuscia, Largo dell'Università snc, 05100 Viterbo (VT), Italy, E-mail address: fbuono@unitus.it

```
                                                                                    Signal peptide
   1                                                                         M  K  M  G  M  L  L  L  V  S  A  L
   1 AAGGACTTCTTGAAATAGCTTCTTCCAGAAGTTGACAGCCAAACAAACTCTGATCATGAAGATGGGGATGCTTCTGCTGGTTTCAGCTCTG

  13  V  L  L  S  A  V  S  P  T  I  A  S  P  S  P  H  H  H  K  N  L  N  I  V  F  D  M  A  Q  K
  92  GTGCTGCTCTCTGCCGTCAGTCCCACCATCGCCAGTCCTTCTCCTCATCATCACAAGAACCTAAACATCGTATTTGACATGGCTCAAAAA

  43  Y  N  E  S  L  S  R  M  Y  F  V  E  D  V  S  S  L  A  D  G  A  N  K  C  Q  D  K  F  F  C
 182  TACAACGAATCTCTCTCTCGGATGTACTTTGTGGAGGACGTGTCGAGTCTGGCTGATGGTGCAAACAAATGTCAGGATAAGTTCTTCTGC

  73  K  V  Y  M  I  L  R  E  H  E  E  L  I  N  R  S  E  E  R  G  L  V  K  N  L  K  K  F  V  D
 272  AAAGTGTACATGATCCTGCGTGAACACGAAGAATTGATTAACCGATCTGAGGAGCGTGGGCTTGTGAAGAACTTGAAGAAATTTGTTGAT

 103  G  I  N  A  N  C  T  E  L  L  K  D  V  V  P  S  D  V  T  K  P  I  P  S  L  L  E  H  L  T
 362  GGCATAAATGCAAACTGTACGGAGTTACTAAAGGATGTGGTCCCTTCAGACGTCACAAAACCGATACCCTCCCTTTTAGAACACCTCACC

 133  R  C  I  Q  S  L  N  M  R  R  S  D  -
 452  CGCTGTATCCAGAGCTTGAACATGAGACGAAGCGATTAATGAACAGACCTGCGACCCGACTGTCAGTGGAGTCCTTGAGAGTTTCATTTT
 542  GTTTTTTGAGAGAATTTCATTTTTTTACATTTTGAAAAACTGTAATGTAATGACTAATAGCAAATTAATGGGGAATATGATTAGATAATA
 632  TGTTGTATACCAGGTTTAAATTGTAAATTTAATTTTTTTCTACGTAATGTCGTTGATAGTTTTTATTTAGGAGTTATCTTCAAGTGAATT
 722  AATATCTGAAGTCCCTCTAGACGATGCACAGCCGTCATAGTTTTGTACTACAAACAACAACACAATGTAATAAATAGTCATTTAACAGAT
 812  ATTTACTTTTATCTGCCCGGTAAGCAGTTTTGAATTAGTACAGTGATGATCACTGTGGTGATCTGAGCACTATCAAATACACCTTCATAT
 902  TTATTCTTTTCAGTATTTAACATAAGTTAAGGCTGTTTTATATATTTGAGTGGCATATTTATTGTAATCCTATCATACAGCATTGAAATA
 992  TGTGTTTACAGAAAAATAAATCGTATTACCCATGAGTGGAGTATATATGGAAATGCTTTGTGCTCACATGTTAATCAGAATTTACAGAAA
1082  GACTTTTTATAACTTTTATTTTTATCTGTTGCATTGATTTTCTTTATGATGCTGTGCTTTTTATCAAGTCATCTTTTCTCATTCAGTCTC
1172  ATTCAACTACTCACACACCTCTTAAATATGTTAAGTTACATTTAAACTGCAGATTATGAGTGCAGCTGAACTACAATTTAACCTGTATCC
1262  ATTTTTTTTTGTTTCACTTTTCATTTCTTTTAAATACATTATATTTGTAGCATTGACCTCATTGCATACTCATTGGCATTAGAATATCTT
1352  TGGTACTCTCAAATTTAATTAGACATCAGTTGATTTAGAGATGGATCAAACCAAGATGTTTGTAATGGATCTAGTTAAAGCACTGCAAAA
1442  CGCAAAAATAATTCAATATAAGAAGGCCAAATGACTCTGAGTTTGTAGAGCACTGGCTGTGATTGTTTGATCATGTCCTTTTCTGTAAGA
1532  CTAGTGGGGAACCACACAATAAAGTATTGGATTTATGTATATCTTTGACTTGCCTTGGACCCAATATTGTACAAGTGTTTAAAACGGCCT
1622  GTTTCTGTTGTAGTTTGCCGTGCTACAACAATAAATGACTATATTGTCAAATCTTTGTATGGATAGATGTTGCCGTGTTCTTTACCCTAG
1712  AGACAGATTGTAACATTGCATATTAAAAATTAAATGTTTATTCAGCTTTTGATCATGATCCACGT
```

Figure S1. The nucleotide sequence and amino acid translation of sea bass IL-4/13A1. Start and stop codons, and in frame stop codon before the main ORF are underlined; ATTTA motifs are in bold and underlined, potential poly(A) signals are boxed; potential N-glycosylation sites are underlined.

```
    CGTCTCATCAACCACACAATAATCAGTCATATCTTATCTTACATTACCGACTGACAGCATTTTTTTCCTTTTTGGTATGAGTGGTAAAGC

 92 TTGGCCGAGGCGTGGGCAATGCAAAAGTATAAAGGTTCTCAGTTGAAGCCTTATAACAAACTGATCAATTCAAGGGCTTTCTGACATAGC
                                                          Signal peptide
  1                                 M  K  M  K  M  L  L  L  V  S  A  V  A  L  L  V  N  S  A  A
182 TTCCTCAAGAGCTCAACAACAGCTTGTATCATGAAGATGAAGATGCTTCTGCTGGTATCTGCTGTGGCTCTGCTGGTAAATTCAGCTGCT

 21  V  S  A  R  P  H  N  V  T  Q  Q  N  L  I  F  D  L  V  E  K  C  I  E  S  R  S  Q  T  F  V
272 GTTAGTGCTCGTCCTCATAACGTTACTCAGCAAAACCTAATATTTGACCTCGTGGAAAAATGCATTGAATCTCGTTCACAGACATTTGTG

 51  D  D  V  S  H  L  A  K  G  S  R  K  C  E  D  R  F  F  C  K  V  H  D  V  L  R  N  T  K  D
362 GATGATGTATCACATCTGGCTAAAGGCAGCAGAAAATGTGAGGATCGGTTCTTTTGTAAAGTGCATGATGTCCTGCGTAACACAAAAGAT

 81  V  C  K  E  E  D  R  K  V  L  V  E  T  L  H  A  Y  N  T  G  R  N  V  Q  C  E  N  T  L  Q
452 GTCTGTAAAGAGGAGGACAGGAAGGTGCTTGTGGAAACCCTGCATGCGTATAATACTGGCAGAAATGTGCAGTGTGAAAATACACTTCAG

111  G  M  T  S  T  G  I  E  I  E  V  S  S  F  L  E  H  V  K  R  C  V  R  H  R  N  F  H  G  T
542 GGAATGACAAGTACAGGCATAGAGATCGAAGTATCCAGCTTTTTGGAACATGTCAAGCGTTGTGTCCGGCACAGAAACTTTCATGGGACC

141  K  K  -
632 AAAAAATAGCAGCTCCTGAATACCACCAAAACCCAATGTGAAGGCTACCAGATACAGCTGCTTGTTTTCTAATTTGTAACTGTATTTAAC

722 ATAAATTATTAAATCTATTTGAATAACATATTTATTGTAATATCATGCAGCATTGAAATACAATTGTACAGAATTTACAGAAAAATAAAA

812 TTGTATTCATTAAGATAAAAAAAAAAAGT
```

Figure S2. The nucleotide sequence and amino acid translation of sea bass IL-4/13A2. Start and stop codons, and in frame stop codon before the main ORF are underlined; ATTTA motifs are in bold and underlined, the potential poly(A) signal is boxed; potential N-glycosylation sites are underlined.

```
                                                                           Signal peptide
  1                                                       M  K  N  F  S  I  Q  S  F  S  V  M  M  M  I  V
  2  CGCTCAACCTGACAGTGCTGGCTCACGGTCTCTTTCAACAACATGAAAAACTTCAGTATTCAGAGTTTCTCAGTGATGATGATGATTGTG

 31   V  V  Y  A  A  S  L  S  Q  H  Q  S  K  R  V  R  Q  K  R  S  H  D  G  C  T  N  A  S  A  N
 92  GTGGTTTATGCAGCATCACTCTCACAACATCAAAGCAAAAGAGTTAGGCAGAAACGAAGTCATGATGGATGTACTAACGCCAGTGCTAAC

 61   N  L  R  H  L  I  H  E  D  A  N  L  M  L  H  N  M  T  E  E  E  N  S  E  M  I  P  W  I  T
182  AATCTCAGACACCTAATTCATGAAGACGCAAATCTGATGTTACACAATATGACAGAAGAGGAAAACAGCGAAATGATTCCATGGATAACT

 91   G  L  K  T  C  V  K  E  F  S  C  L  A  E  K  A  L  N  Q  S  K  N  H  K  L  K  R  L  T  H
272  GGATTAAAAACATGTGTGAAAGAGTTCTCCTGTCTCGCTGAGAAGGCTCTGAACCAAAGCAAAAACCACAAATTAAAAAGACTCACCCAT

121   H  L  H  Q  Y  N  K  K  L  N  E  T  D  C  H  L  K  E  H  P  K  C  T  V  H  R  V  L  E  D
362  CACTTGCATCAGTACAACAAGAAGCTCAATGAGACAGACTGTCACCTGAAGGAGCACCCCAAATGTACTGTGCACAGAGTTCTTGAGGAC

151   I  K  T  C  I  V  K  C  P  G  K  N  -
452  ATCAAGACATGTATTGTCAAGTGTCCTGGGAAAAACTGAGCAATCACCCCACAAATTATTACGAGTGATCACGTGTTATTTAAGTAAATT

542  ATTCTATTTATCGTGTGCCCAGTTGGCTCAGTGGGTAGAGCTGGCAACCCACGTACCGAGGCTGAGTCCTTGCTGC
```

Figure S3. The nucleotide sequence and amino acid translation of sea bass IL-4/13B. Start and stop codons, and in frame stop codon before the main ORF are underlined; ATTTA motifs are in bold and underlined; potential N-glycosylation sites are underlined.

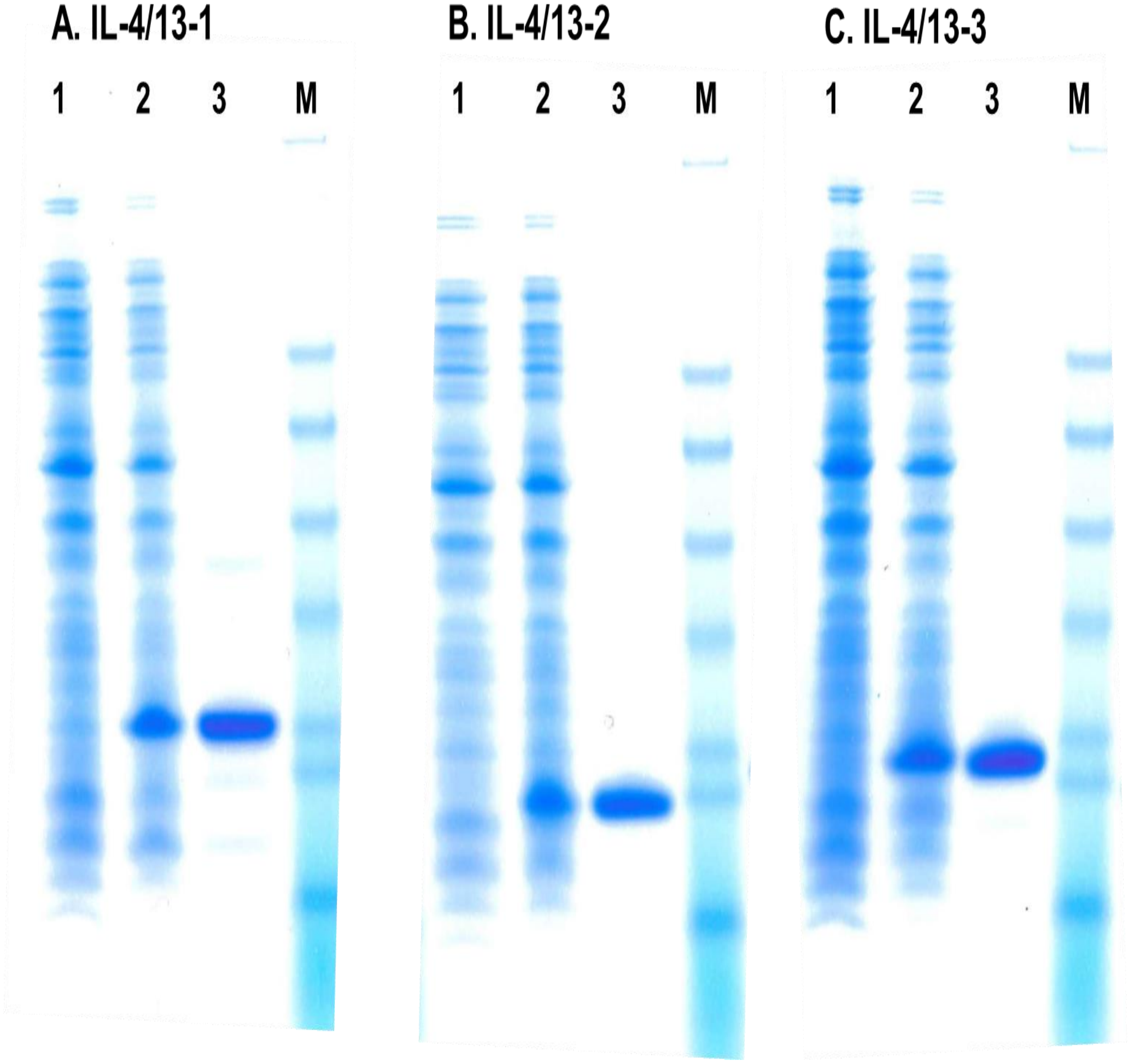

Figure S4. SDS-PAGE analysis of seabass rIL-4/13 isoforms expression and purification from *E. coli* BL21 Star (DE3). The SDS-PAGE gel was stained with SeeBlue (Invitrogen). Samples loaded in the different lanes: 1: a sample from un-induced BL21 cells; 2: BL21 transformed by IL-4/13-expressing plasmid and induced with 1 mM IPTG for 4 h; 3: expression product purified from transformed cells expressing IL-4/13 isoforms; M: Protein marker, SeeBlue (Invitrogen); IL-4/13-1 = IL-4/13B; IL-4/13-2 = IL-4/13-A1; IL-4/13-3 = IL-4/13A2.
